# Editorial: Ovarian Ageing: Pathophysiology and Recent Development of Maintaining Ovarian Reserve

**DOI:** 10.3389/fendo.2020.591764

**Published:** 2020-09-23

**Authors:** Kazuhiro Kawamara, Tom Kelsey, Osamu Hiraike

**Affiliations:** ^1^Advanced Reproductive Medicine Research Center, International University of Health and Welfare (IUHW), Otawara, Japan; ^2^School of Computer Science, University of St Andrews, St Andrews, United Kingdom; ^3^Department of Obstetrics and Gynecology, Graduate School of Medicine, University of Tokyo, Tokyo, Japan

**Keywords:** ART, IVF, AMH, FSH, ovary, oocyte, PCOS

Increased understanding of ovarian aging is essential in the modern world. Ages at menopause have remained stable at average of 51 years in Europe and 49 years in China, with some evidence that environmental xenobiotic toxicity is reducing reproductive lifespans for women. Most women have 12% of their follicle pool remaining at age 30, falling to 3% at age 40 years. However, age at motherhood has increased steadily over the last few decades, with, for example, birth rates for ages 35–39 and 40–44 years more than doubling in the United States of America between 1990 and 2015. Since 1970 the average maternal age at birth in most OECD countries has increased by 2–5 years. The adverse effects of increased maternal age on reproductive outcomes are well-known, with maternal age being the strongest predictor of *in vitro* fertilization (IVF) success, and a decline in female fecundity with age associated with a reduction in oocyte quality and an increased risk of miscarriage. Hence there is a clear potential conflict between the biology of reproduction working against motherhood later in life, and the societal expectation of motherhood at an age convenient for the mother in terms of education, career, relationship, and family considerations. The postponement of starting a family therefore has implications on the ability of women to conceive and parents to produce additional offspring. Societal changes in terms of policy interventions regarding career breaks and higher institutional gender equity can be useful, as can efforts to counter scarce information or poor understanding such as the diffuse idea of omnipotence of modern medicine. But the burden of effort in maintaining ovarian reserve, and hence fertility, remains with academic and clinical teams, working together to optimize fertility outcomes, based on careful analyses of high-quality data to identify the evidence needed to underpin safe and reliable clinical practice. It should also be noted that ovarian aging can occur as a result of chemo- and/or radiotherapies used to treat cancer patients of any age, and hence that the lessons learned from studies into ovarian aging are useful and applicable not only for spontaneous pregnancies and assisted conception cycles, but also oncofertility-related pregnancies.

We asked the contributors of this Research Topic to provide useful insights into the underlying mechanisms of fertility, with particular emphasis on endocrine factors. Whilst animal models were not excluded, we encouraged submissions based on human studies and that therefore have direct relevance to the unique challenges facing our species. The result is a varied yet comprehensive collection of five articles and two abstracts.

Follicle–stimulating hormone (FSH) was identified in the 1980s as an ovarian factor that reduced gonadotropin-releasing hormone (GnRH)-induced luteinizing hormone (LH) secretion, and swiftly became the standard approach used to stimulate gonadal function in hypogonadotropic hypogonadism. FSH entered clinical use in controlled ovarian stimulation as a stimulant for follicular growth in assisted reproduction. However, robust evidence that supports the clinical use of FSH is still sparse. Abbara, Patel et al. designed and reported on a study aimed to evaluate FSH requirements for follicular growth during controlled ovarian stimulation. Initial recombinant FSH (rFSH) doses were adjusted for body weight and matched to the follicle sizes at time of oocyte maturation trigger. Their key finding is that an insufficient weight-adjusted dose (i.e., requiring significant dose increase after an initial dose) led to variability in follicle sizes, hence to fewer mature oocytes being obtained with clear negative impact on the chances of a live birth from the cycle. Given that the literature suggests an upper threshold exists for useful FSH dosing, this study provides IVF practitioners with lower limits in addition to upper limits for optimal dosing regimen for individual cycles. The crucial relationship between follicle profile at time of trigger and mature oocytes obtained was established by the same research group in 2018.

A well-established issue with IVF is that standard *in-vitro* culture conditions exert oxidative stress, with the presence reactive oxygen species (ROS) resulting in an imbalance between oxidants and antioxidants, potentially leading to structural and functional changes in cells. Sasaki et al. reviewed the underlying mechanisms of the age-related decline in oocyte quality, with specific focus on oxidative stress. They identify the primary mechanisms involved, describe the vulnerability of oocytes to these mechanisms, and suggest possible therapeutic strategies to reduce the adverse effects of oxidative stress on ovarian aging.

The utility of growth hormone (GH) in assisted conception is extensively reviewed in seperate articles by Blumenfeld and Hart with both authors identifying the lack of power in existing studies that reduces our confidence in the claimed benefits of routine GH treatment, even for those cases that are classified as poor responding according to the Bologna definition. Hart emphasizes the key point that no meta-analysis has shown that the use of GH increases the chances of live birth, which is, of course, the primary desired outcome. His detailed identification and description of the key literature in this area highlights the use of GH as one of several “folklore” methods in assisted conception, used in line with prevailing clinical procedural norms, rather than used as result of detailed supporting evidence. Blumenfeld makes many of similar deductions, but additionally addresses the issue of what treatments should be used if GH is found not to be suitable. Interestingly, GH is not ruled out with the suggestion that it may be effective in exceptional and restricted cases—poor ovarian response patients with borderline GH deficiency.

Kisspeptins are proteins encoded by the KISS1 gene in humans. KISS1 was originally identified as a human metastasis suppressor gene. Kisspeptins were later found to have an important role in initiating secretion of GnRH at puberty, and as a method to stimulate ovulation in IVF cycles leading in principle to a more physiological increase in reproductive hormones and oocyte maturation during IVF treatment. Trevisan et al. (accepted) performed a detailed and comprehensive comparative analysis of KISS1 polymorphisms with respect to key factors that influence the prospects of live birth, finding that the KISS1 rs1132506 polymorphism was associated with decreased AMH levels, increased estradiol and prolactin levels and decreased number of mature oocytes and embryos.

Amenorrhea is a key characteristic of perimenopause and the aging ovary. Anti-Mullerian Hormone (AMH) is now accepted as a biomarker for both ovarian activity—and hence remaining ovarian reserve—and as a diagnostic predictor of polycystic ovarian syndrome (PCOS). Abbara, Eng et al. report that AMH is a strong predictor for menstrual disturbance due to PCOS, and furthermore that the risk of menstrual disturbance increases with elevation in serum AMH. In addition to these main findings, the authors show that high AMH leads to more LH in follicular phase gonadotropin levels, commensurate with an increase in GnRH pulsatility. Study participants with more LH-predominant gonadotropin secretion also had increased rates of menstrual disturbance. These findings increase our understanding of the complex and dynamic endocrine and paracrine milieu associated with the aging ovary.

## Concluding Remarks

Taken together, the research studies and reviews contained in this Research Topic provide clear and detailed insights into several fundamental aspects of the important and challenging undertaking of maximizing the chances of safe live births for the increasing number of women seeking to delay motherhood. The results are useful to clinical teams working in assisted conception, and also indicate to research groups the challenges that remain to gain a fuller understanding of ovarian aging.

## Author Contributions

All authors listed have made a substantial, direct and intellectual contribution to the work, and approved it for publication.

## Conflict of Interest

The authors declare that the research was conducted in the absence of any commercial or financial relationships that could be construed as a potential conflict of interest.

